# Aetiologic diagnosis of coronary artery aneurysm in a patient with pancreatitis, dacryoadenitis, and sialadenitis

**DOI:** 10.1093/eurheartj/ehac536

**Published:** 2022-10-01

**Authors:** Antonio Iaconelli, Giuseppe Rovere, Domenico D'Amario, Mattia Galli, Tommaso Sanna

**Affiliations:** Fondazione Policlinico Universitario Agostino Gemelli IRCCS, Largo Francesco Vito, 1–00168 Rome, Italy; School of Cardiovascular and Metabolic Health, University of Glasgow, Glasgow, UK; Fondazione Policlinico Universitario Agostino Gemelli IRCCS, Largo Francesco Vito, 1–00168 Rome, Italy; Fondazione Policlinico Universitario Agostino Gemelli IRCCS, Largo Francesco Vito, 1–00168 Rome, Italy; Department of Cardiovascular Sciences, Catholic University of the Sacred Heart (UCSC), Rome, Italy; Department of Cardiovascular Sciences, Catholic University of the Sacred Heart (UCSC), Rome, Italy; Maria Cecilia Hospital, GVM Care and Research, Cotignola, Italy; Fondazione Policlinico Universitario Agostino Gemelli IRCCS, Largo Francesco Vito, 1–00168 Rome, Italy; Dipartimento di Scienze Cardiovascolari, Università Cattolica del Sacro Cuore (UCSC), Rome, Italy

A 67-year-old man was admitted to our cardiology ward for a diagnostic work-up.

Coronary angiography (*Panels A and B*) and multidetector computed tomography coronary angiography (CTCA, *Panels C and D*) revealed an aneurysm of the proximal segment of the left anterior descending artery.

**Figure:**
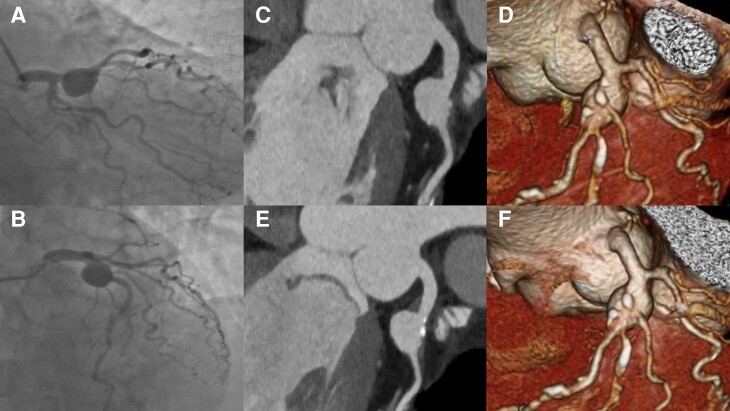


Coronary artery aneurysm (CAA) is an infrequent finding and an aetiologic diagnosis is crucial for the treatment selection.^1^ Our patient reported a 10-year history of lymphadenopathy and recurrent episodes of sialadenitis, dacryoadenitis, prostatitis and pancreatitis. Prior biopsies of bone marrow, lymph nodes, prostate, salivary glands and pancreas showed lymphoplasmacytic infiltrates suggestive of an immune-mediated disease but a specific diagnosis had not been made.

A comprehensive diagnostic work-up revealed elevated IgG4 levels (440 mg/dL; n.v. ≤ 135 mg/dL) that, according to medical history and histological findings, confirmed the diagnosis of IgG4-related disease (IgG4-RD).

The patient was treated with corticosteroids and anticoagulants. One-year follow-up CTCA showed unchanged findings (*Panels E and F*), highlighting the importance to consider conservative approaches for this condition.

IgG4-RD often presents with pancreatitis, dacryoadenitis and sialadenitis but clinical manifestations are recognized in every organ system, including coronary arteries. Glucocorticoids are the first-line treatment of IgG4-RD, potentially resulting in disease remission.^2^ The history of pancreatitis, sialadenitis, dacryoadenitis and lymphadenopathy in a patient with CAA should alert cardiologists to suspect IgG4-RD.

**Guarantor:** The scientific guarantor of this publication is Antonio Iaconelli as corresponding author.

**Statistics and biometry:** No complex statistical methods were necessary for this paper.

**Informed consent:** Written informed consent was obtained from the patient involved in this study.

**Methodology:** Case.

**Funding:** None declared.
